# Osimertinib in Combination with Bevacizumab Fails in Advanced Lung Adenocarcinoma Harboring EGFR T790M

**DOI:** 10.1055/s-0041-1731067

**Published:** 2021-06-26

**Authors:** Xiaoying Zhang

**Affiliations:** 1 Department of Geriatrics, Qilu Hospital of Shandong University, Jinan, People's Republic of China

Lung cancer has been the most frequently diagnosed cancer and the leading cause of cancer-related death in the world for several decades. 1 In 2018, more than one-third of all new lung cancer cases and nearly half of all lung cancer deaths in the world occurred in China, 2 representing a large economic and humanistic burden for Chinese people. 3 Non–small cell lung cancer (NSCLC) is the most frequent subtype of lung cancer which usually has potent oncogenic driver gene mutations, such as epidermal growth factor receptor (EGFR) mutations. Currently, major international clinical recommend EGFR-tyrosine kinase inhibitors (TKIs) as first-line treatment for EGFR-mutant NSCLC. Indeed, this therapeutic strategy has significantly improved the progression-free survival (PFS) of NSCLC patients. However, most patients treated with EGFR TKIs develop acquired resistance and thereby experience disease progression after a median period of 10 to 13 months. 4 The molecular mechanisms of TKI resistance can be classified as three types, including EGFR mutation, activation of adaptive signaling pathways, and histological transformation. 5 Previous study revealed that approximately 50% of NSCLC cases developed TKI-resistance acquired EGFR Thr790Met (EGFR T790M) mutation. 6

Osimertinib is a third-generation EGFR-TKI. So far, it is the only EGFR-TKI capable of overcoming NSCLC with EGFR T790M mutation, but the median PFS with osimertinib monotherapy was still less than 13 months. 7 Therefore, new combination strategies must be adopted to improve tolerance and delay disease progression. Preclinical study reported that EGFR-TKI plus antivascular endothelial growth factor (VEGF) inhibitor could result in a synergistic effect in xenograft models harboring EGFR T790M mutation. 8 Bevacizumab, an anti-VEGF-A monoclonal antibody, is an essential component in the treatment of NSCLC patients. However, the clinical efficacy and safety of osimertinib plus bevacizumab has not yet been established for the treatment of EGFR-mutant NSCLC.

In a study recently published in JAMA Oncology, titled “Efficacy of Osimertinib Plus Bevacizumab vs Osimertinib in Patients With EGFR T790M-Mutated Non-Small Cell Lung Cancer Previously Treated With Epidermal Growth Factor Receptor-Tyrosine Kinase Inhibitor West Japan Oncology Group 8715L Phase 2 Randomized Clinical Trial,” by Akamatsu et al 9 enrolled 81 advanced NSCLC patients who acquired EGFR T790M mutation after receiving prior EGFR-TKI treatment and conducted a phase-II randomized clinical trial in Japan to evaluate the safety and efficacy of osimertinib in combination with bevacizumab. In this study, the patients were randomly assigned to an osimertinib group ( *n* = 41; treated with osimertinib alone) or a combination group ( *n* = 40; treated with osimertinib plus bevacizumab) between August 2017 and September 2018. The median age of the enrolled patients was 68 years (range: 41–82 years). Among them, 37 (45.7%) patients had the Eastern Cooperative Oncology Group (ECOG) performance status of 0 and 21 (25.9%) developed metastasis. The overall response rate (ORR) of combination group was better than that of osimertinib group (68 vs. 54%). The median PFS in the osimertinib arm was 13.5 months and those in the combination arm was 9.4 months. Therefore, no statistically significant difference in median PFS between two groups was observed (adjusted hazard ratio = 1.44; 80% confidence interval = 1.00–2.08; *p* = 0.20). Similarly, median overall survival (OS) wasn't longer with osimertinib plus bevacizumab (not reached in the combination group vs. 22.1 months in osimertinib group; *p* = 0.96). However, the median time to treatment failure in combination group was shorter as compared with that in osimertinib group (8.4 vs. 11.2 months; *p* = 0.12). Common severe adverse events (≥grades 3) observed in the combination group were proteinuria ( *n* =  9) and hypertension ( *n* =  8).

This is the first randomized phase-II trial to explore the efficacy of adding anti-VEGF inhibitor to osimertinib. Unfortunately, the observations indicated that osimertinib in combination with bevacizumab failed to show prolongation of PFS and OS as compared with osimertinib monotherapy in NSCLC patients with EGFR T790M mutation, although the ORR was slightly better and the cancer therapy-related toxicities were tolerable. In contrast, previous studies have reported that one randomized phase-II and two phase-III trials reported that first-generation EGFR TKI (erlotinib) plus anti-VEGF inhibitor showed more benefits in PFS as compared with EGFR TKI alone. 10 11 12 Two single-arm clinical trials reported the preliminary results of osimertinib plus anti-VEGF inhibitor just recently. The study conducted by Yu et al 13 reported that osimertinib plus ramucirumab demonstrated a median PFS of 11.0 months and an ORR of 76% among 25 NSCLC patients harboring EGFR T790M mutation. Another clinical trial of osimertinib plus bevacizumab in 49 NSCLC patients with EGFR sensitizing variants conducted by Yu et al 14 showed a median PFS of 18.4 months and an ORR of 80%. However, considering the PFS of osimertinib monotherapy in pivotal studies was 10.1 months in EGFR T790M-mutated NSCLC and 18.9 months among EGFR-TKI–naive NSCLC patients, 7 the results of these two clinical trials were not so intriguing. Since the present study adopted a randomized study design, the results of the present study are more reliable than the previous single-arm trials.

As mentioned above, the present study and the previous studies have failed to show the advantages of osimertinib plus anti-VEGF inhibitors. One possible explanation for this situation is that prior treatment exposure and tumor regrowth may lead to changes in tumor microenvironment and thereby prevent synergy between osimertinib and bevacizumab or lead to resistance to anti-VEGF inhibitor. As the present study did not collect any blood or tissue samples before and after treatment, none potential mechanism of bevacizumab resistance can be further explored and discussed.

This clinical trial has several limitations. First, the number of enrolled patients was quite small. Therefore, results of this study would not be 100% conclusive. However, the findings of this study draw attention to the development of EGFR TKI plus anti-VEGF inhibitors in treatment of NSCLC. Indeed, several research groups are currently conducting larger-scale studies. Second, compared with global studies of chemotherapy plus anti-VEGF inhibitors, proteinuria incidence in Japanese studies was much higher (26 vs. 4%). 15 Racial difference may seriously affect the feasibility of bevacizumab in Japanese population.

Despite the limitations mentioned above, the present study provides useful information. The data of the present study directly affects the choice of first-line regimen in NSCLC patients with EGFR mutations. It suggested that osimertinib in combination with bevacizumab is no longer effective in NSCLC patients with EGFR T790M mutation. Additionally, prior exposure to anti-VEGF inhibitors could adversely affect second-line treatment with osimertinib plus bevacizumab.
